# Notes and Miscellany

**Published:** 1909-08

**Authors:** 


### Notes and Miscellany.

"A TAX on print paper is a tax on intelligence, a shade on the lamp of enlightenment, and a cloud over the sun of civilization."—Remarks of Hon. Thomas P. Gore, of Oklahoma, in the Senate of the United States.

---

DR. WM. F. DREWRY HONORED.—The American Medico-Psychological Association, at its recent session in Atlantic City, N. J., elected by acclamation Dr. Wm. F. Drewry, Superintendent Central State Hospital, Petersburg, Virginia, President for the ensuing year. This is regarded by insanity experts, alienists, etc., as one of the highest honors in the profession.

---

DR. MATAS HONORED.—At the meeting of the American Surgical Association, held in Philadelphia the first week in June, Dr. Rudolph Matas, head of the Department of Surgery of the Tulane University, was elected President for the year 1909-10. The JOURNAL joins Dr. Matas' many friends in congratulating him on this deserved recognition.

---

A LESS MURDEROUS FOURTH.—The Independence Day celebration this year was much more quiet and freer from casualties than any Fourth of July for many years, and this in spite of the fact that it was observed on two days. The returns on Tuesday morning showed only sixty-one deaths, eleven less than last year, and 3246 more or less serious injuries. The cases of tetanus resulting from these will, it is hoped, be fewer, owing to the more general employment of tetanus antitoxin as a prophylactic.—*New York Medical Record.*

---

DR. G. W. BASKETT, of Van Alstyne, Texas, has purchased the interest Dr. G. P. Baskin has in the Texas Sanitarium at Llano. Dr. Baskett has gone to Llano with his family, and is the Medical Director of the Texas Sanitarium. He is a middle-aged physician of experience and position, good judgment, and splendid business qualifications. We predict a wonderful improvement in the services at the Sanitarium under the management of Dr. Baskett. The Doctor, by the way, has two sons who are graduates of medicine from the University of Michigan, both holding hospital appointments in the North at the present time.

---

HAY FEVER is an octupus against which the Gatling guns of Materia Medica have been fiercely fired for many years. There is a new "gun" on the market. Our front cover tells just enough about it to whet our mental appetite for more light. That's a rather mixed metaphor—but let that pass. The continuous antiseptic spray plan of Hay Fever medication by means of Nazeptic Wool has the ring of common sense any way. Our old conservative Baltimore friends never indulge in hyperbole in their advertising chats in the "Red Back." What they say usually "goes" in Texas because they have never abused our confidence. We have a sample of Nazeptic Wool on our editorial desk as we write this, and it certainly "looks good" to us. There is a package in Baltimore for every member of the "Red Back" family. Have you sent for yours?—Written especially for the TEXAS MEDICAL JOURNAL.

---

THE DEADLY FLY.—To warn the people of the dangers of flies, and to show them how to get rid of the pests, the Chicago Health Department has issued a bulletin, in which the pesky nuisances are called all sorts of bad names. "Flies are the dirtiest and filthiest of vermin," the bulletin says. "They are born in filth, live in filth and carry filth around with them. Millions of death-dealing germs cling to them, only to be scattered upon those whom they touch. Now is the time to build your lines of defense. Prepare to fight them as you would wild beasts seeking your life." A good fly poison, not dangerous to human life, the bulletin adds, is a solution of bichromate of potash, one dram dissolved in two ounces of water, and sweetened with a little sugar. Put some in shallow dishes and place throughout the house. Another is cobalt chloride, one dram dissolved in three ounces of water, placed in shallow dishes as above. To clear rooms in which there are large numbers of flies burn pyrethrum powder or blow black flag into the air of the room. These do not kill the flies; they are merely stunned and fall to the floor. They must then be gathered up and destroyed.—*Exchange.*

---

THE bibliography apology for plagiarism enables a medical ass to pose for a gifted scientist. Did you ever hear of any one investigating bibliography?

Medical mathematics: The treatment of chronic disease represents *a majority of medical practice.* To this subject leading textbooks devote approximately 1-80 of their total discussion, leading (so rated) medical journals 1-400, other journals 1-100, medical colleges 1-500, and prominent medical societies less than any. Again I repeat, *for what are we practicing medicine?*

Each year the leading medical journals (orthodox criterion) print volumes of self-advertisements of surgeons and specialists (such as Dr. Blank's series of 200 appendectomies without a death), and *without the slightest investigation,* but not a line favorable to those proprietary innovations which are open to the fullest investigation, and have survived the severest tests of merit.

The most important work for those investigating remedial agents has not yet been started or even suggested, namely, a determination of the accuracy of the recognized text-book physiological actions of official drugs. Exhaustive work in this direction would doubtless weed out many useless agents and dignify many useful ones. It would at least annihilate the drugless therapists, and incite conscientious physicians to resume the study of medical materials. If the orthodox physiological actions are accurate our latter-day therapeutic negativeness is inexcusable. Otherwise we are just plain dupes of pseudo science.

The notoriously scant attendance at medical society meetings and the "skimming" of medical journals is partly justified. The average medical paper could be cut one-half by eliminating useless verbiage, circumlocution, tautology, redundancy, and unnecessary bibliography, statistical details and axiomatic truths. As regards results a majority of medical contributions are a waste either of wind or space. A time limit on papers, and an intelligent, snappy editorial blue pencil would doubtless popularize the two greatest means of post-graduate education.—*Bulletin of Animal Therapy.*

